# A prenatal missed diagnosed case of submicroscopic chromosomal abnormalities by karyotyping: the clinical utility of array-based CGH in prenatal diagnostics

**DOI:** 10.1186/1755-8166-7-26

**Published:** 2014-04-15

**Authors:** Aihua Yin, Jian Lu, Chang Liu, Li Guo, Jing Wu, Mingqin Mai, Yanfang Zhong, Xiaozhuang Zhang

**Affiliations:** 1 Prenatal Diagnosis Centre, Guangdong Women and Children Hospital, Guangzhou, Guangdong 510010, China; 2 Maternal and Children Metabolic-Genetic Key Laboratory, Guangdong Women and Children Hospital, Guangzhou, Guangdong 510010, China

**Keywords:** Array-based CGH, Prenatal diagnostics, Submicroscopic chromosomal abnormalities

## Abstract

**Background:**

Array-based comparative genomic hybridization possesses a number of significant advantages over conventional cytogenetic and other molecular cytogenetic techniques, providing a sensitive and comprehensive detection platform for unexpected imbalances in the genome wide.

**Case presentation:**

The newborn proband, demonstrated with craniofacial dysmorphism and multiple malformations, was born to a family with spontaneous abortions. This pregnancy was uneventful, except the prenatal ultrasound examination showed an increased nuchal translucency at 12^+^ weeks of gestation. Cytogenetics revealed an apparently normal karyotype, and the couple decided to continue the pregnancy. Array-based CGH analysis was applied to the affected infant, identified a combination of 18p deletion and 7q duplication. Further study indicates that the unbalanced translocation was inherited from a balanced translocation carrier parent.

**Conclusions:**

In review of the case, several overlooked points leading to the missed diagnosis should be discussed and certain quality control strategies should be adopted to avoid similar problems in the future. Array-based CGH and karyotyping techniques are complemented by diverse detection spectrum and resolutions, and a combination of these methods could help providing optimal genetic diagnosis. Given that the array-CGH analysis will not introduce additional risk to patients, it is reasonable to recommend those already undergoing invasive testing should take array-based CGH as an adjunct to conventional cytogenetic tests and other molecular cytogenetic analysis.

## Background

Karyotyping is the predominant technique for prenatal diagnosis of chromosomal abnormalities, but most chromosome banding techniques are time-consuming and limited to resolutions of 5 to 10 Mb [1,2]. As prior studies indicated, conventional karyotyping only identify chromosomal anomalies in about 35% of pregnancies with fetal ultrasound abnormalities, depending on the types of these anomalies [3]. Molecular cytogenetic techniques such as fluorescent in situ hybridisation (FISH), quantitative fluorescent PCR (QF-PCR) and multiplex ligation-dependent probe amplification (MLPA) overcome some of those limitations and are used as adjuncts to conventional methods for detecting common chromosome numerical anomalies [1], but none of them provides a genome wide screening for unexpected imbalances [4]. Fortunately, array-based comparative genomic hybridization (array-based CGH) technology can simultaneously evaluate regions across the entire genome and allowed for detection of unbalanced structural and numerical chromosome abnormalities of less than 100 kb [5]. The array-based CGH platform used for clinical prenatal diagnosis is able to enhance the detection rate by 10 to 16 percent of pregnancies with fetal ultrasound anomalies but not detected by conventional cytogenetic or other molecular cytogenetic techniques [6-9].

In this report, we present a prenatal missed diagnosed case of submicroscopic chromosomal abnormalities by karyotyping, and try to demonstrate the clinical utility of array-based CGH in prenatal diagnostics by providing a retrospective analysis of those overlooked points leading to the missed diagnosis of the case.

## Case presentation

### Clinical description

**Figure 1:**
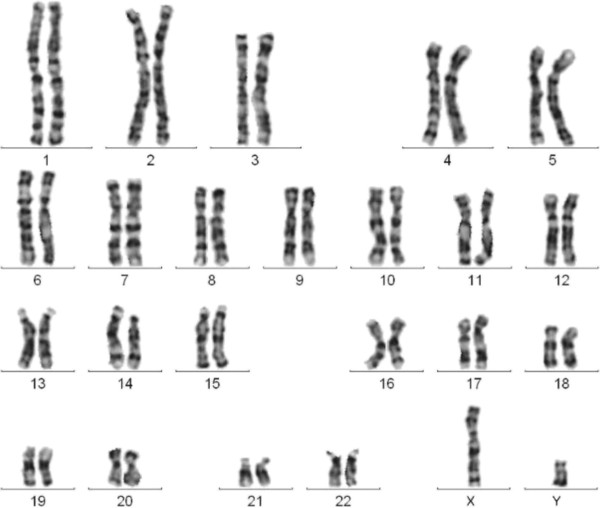
**GTG banded karyotype of the fetus.** The karyotype revealed a apparently normal karyotype 46,XY at a resolution of 400 bands.

The proband, a 1-day-old boy, was the first child of non-consanguineous, healthy 35 year-old parents. The couple had a past history of twice spontaneous abortions both occurred between 7 and 10 weeks of gestation, but product-of-conception (POC) samples were not analyzed. The cytogenetic analyses of the couple preformed at another hospital revealed apparent normal karyotypes. This pregnancy was uneventful, and there was no prenatal exposure to teratogens. However, the prenatal ultrasound examination showed an increased nuchal translucency (4.0 mm) at 12^+^ weeks of gestation. Taking into account of the advanced maternal age as well, interventional prenatal diagnosis was referred to the couple. After understanding detailed information about sampling procedures, the risk of fetal mortality and the limitations of the testing, the couple decided to receive an amniocentesis at 18 weeks of gestation for further diagnosis. Amniotic fluid cells were cultured in situ and their karyotypes were analyzed by using G-banding technique [10], the cytogenetics revealed a apparently normal karyotype 46,XY (Figure 1).

**Figure 2:**
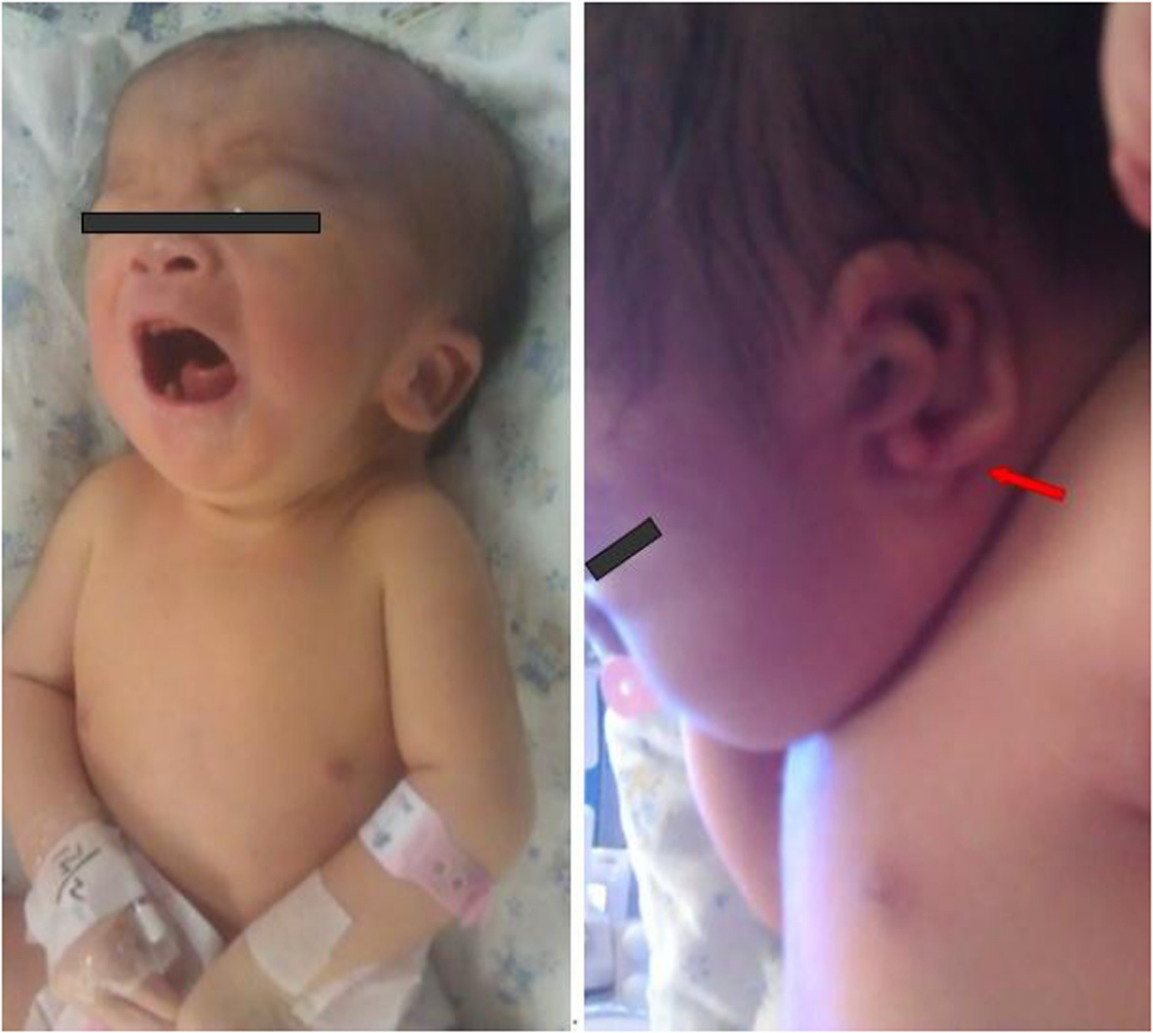
**Clinical features of the newborn proband.** The proband was born with skull joint separation, round face, low-set and dysplastic ears.

Following ultrasound examinations showed an increased size in dimensions of biparietal diameter and abdominal circumference, fetal cerebral ventriculomegaly, increased nuchal folder, polyhydramnios. However, given their past history of spontaneous abortions, the couple was eager to have children, and since the cytogenetic analysis result of this fetus was negative, they decided to continue the pregnancy. The proband was born at 39 weeks and 2 days of gestation by vaginal delivery. His birth weight was 2,688 g (25th centile), length was 47.5 cm (25th centile), and head circumference was 33.5 cm (75th centile). Apgar scores of the infant were 8 at 1 min and 8 at 5 min. Notable physical features included skull joint separation, single transverse palmar crease on both hands, craniofacial dysmorphism including round face, low-set and dysplastic ears (Figure 2). Ultrasonic tests of brain/abdomen and cardiovascular examination were postponed according to the parents of the affected infant.

### Cytogenetic analysis

Peripheral blood samples were obtained from the proband and his parents for cytogenetic analyses. Sample collection procedures were approved by Guangdong Women and Children Hospital Institutional Review Board and Ethics Committee of Guangzhou Medical College, China. The family gave their written informed consent. Peripheral blood samples were cultured for 72 hours in RPMI medium, after that metaphase chromosomes were analyzed by the standard G-banding technique.

**Figure 3:**
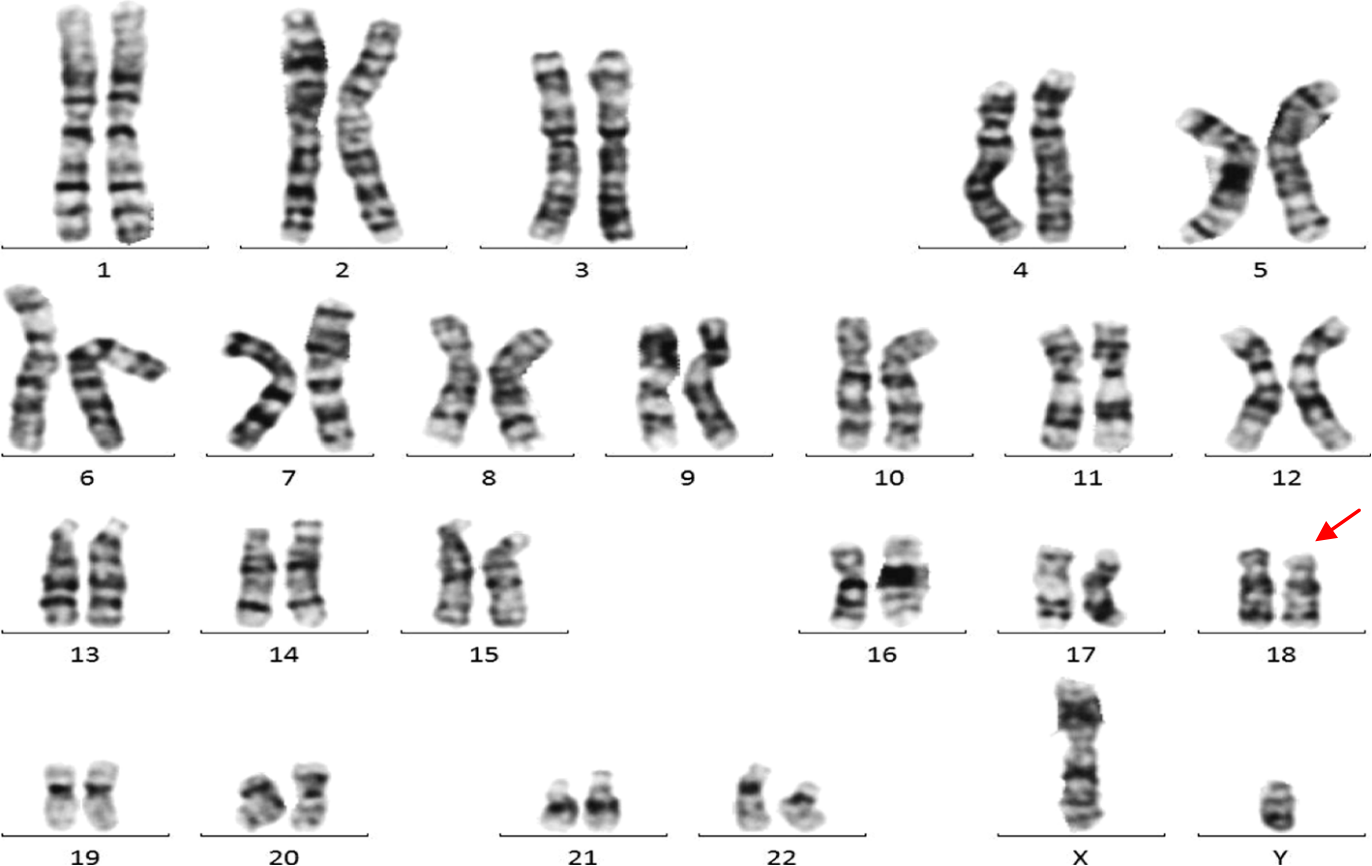
**GTG banded karyotype of the proband.** The proband indicated a possible abnormal karyotype [46,XY,?del(18)(p11.3)].

The cytogenetic analysis of the peripheral blood sample from the proband initially revealed a possible chromosome 18p deletion, but the breakpoint and the deletion region were difficult to determine with the limited resolution of chromosome banding technique (Figure 3).

### Array-based CGH analysis

Genomic DNA from the proband was isolated from the whole blood by standard procedures using Fuji DNA Blood Mini kit. Array-based CGH analysis was performed using Agilent’s 8 × 60 K commercial arrays (Agilent Technologies, CA, USA), which consist of 60,000 oligonucleotide probes and evaluate the whole genome with an effective backbone resolution of roughly 50 Kb. 500 ng of experimental and gender-matched reference DNAs (Promega, Madison, WI) were digested with *Alu* I and *Rsa* I restriction endonucleases (Promega) and fluorescently labeled with cyanine 3-dUTP and cyanine 5-dUTP respectively. Labeled experimental and reference DNAs were purified, combined, denatured, pre-annealed, and hybridized to the microarrays in a rotating oven (20 rpm) at 65°C for 24 hours. The data was analyzed with Agilent Genomic Workbench Lite Edition 6.5.0.18 software (Agilent Technologies). Aberrations were identified using the Agilent Genomic Workbench Lite software via the Aberration Detection Method-2 algorithm with a sensitivity threshold of 6.0 and a data filter that rejected aberrations that did not include at least five probes with a log_2_ set of 0.25. All quality control metrics passed.

**Figure 4:**
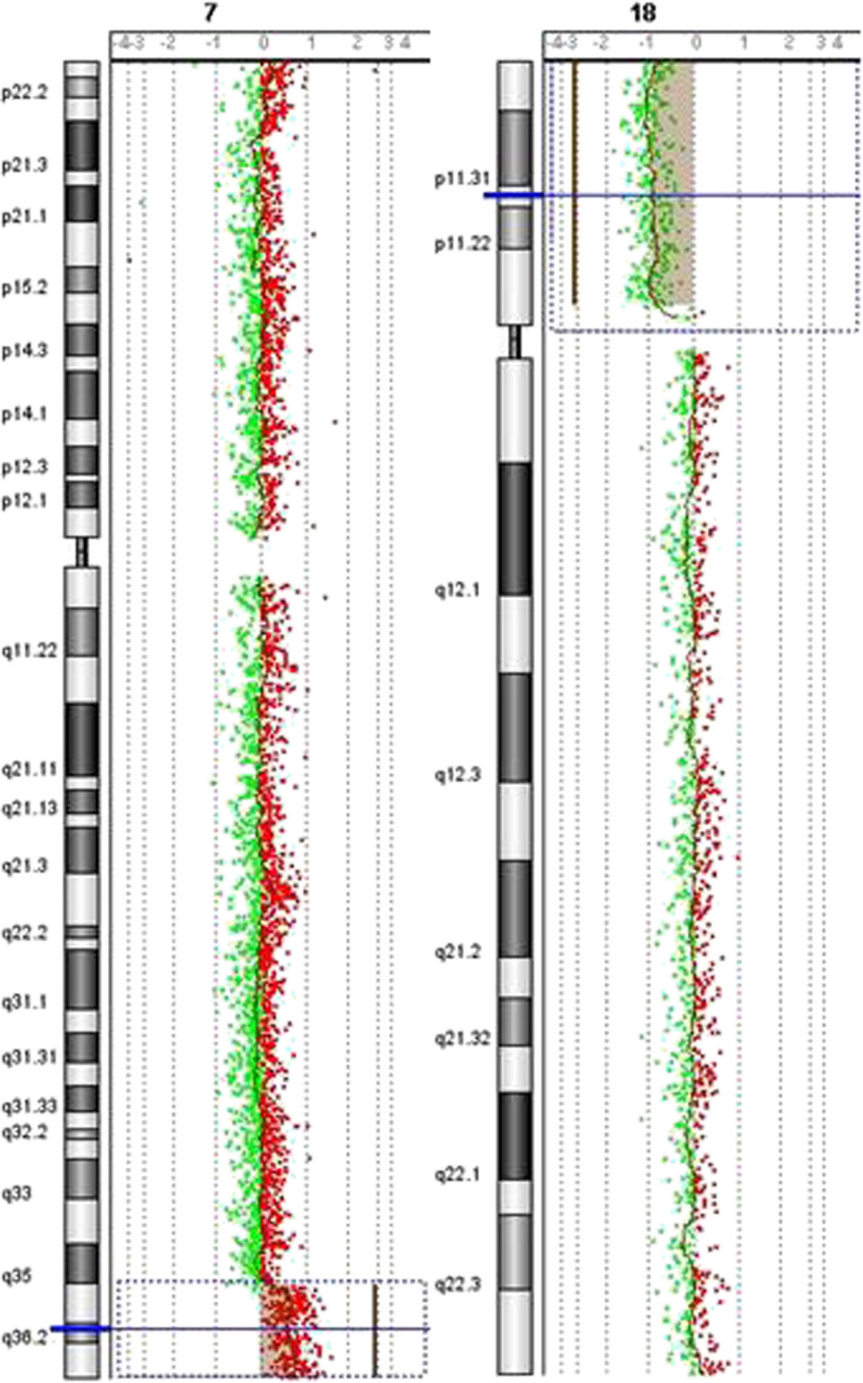
**Array-CGH analysis results of the proband.** The anaylsis indicated a combined of 7q duplication and 18p deletion in the proband.

As shown in Figure 4, a combination of 18p deletion and 7q duplication was identified in the proband by array-CGH analysis. A deletion spanning approximately 14 Mb was detected at 18p11.32-p11.21, with the deleted base pair coordinate ranging from 4,316 to 14,216,904 (hg18). Array-based CGH analysis also identified a duplication of about 11.2 Mb that involving the 7q36.1- q36.3 region, with distal breakpoint falling between 147,580,628 bp (last duplicated oligomer) and 158,781,397 bp (first normal oligomer).

### Origin of the translocation

**Figure 5:**
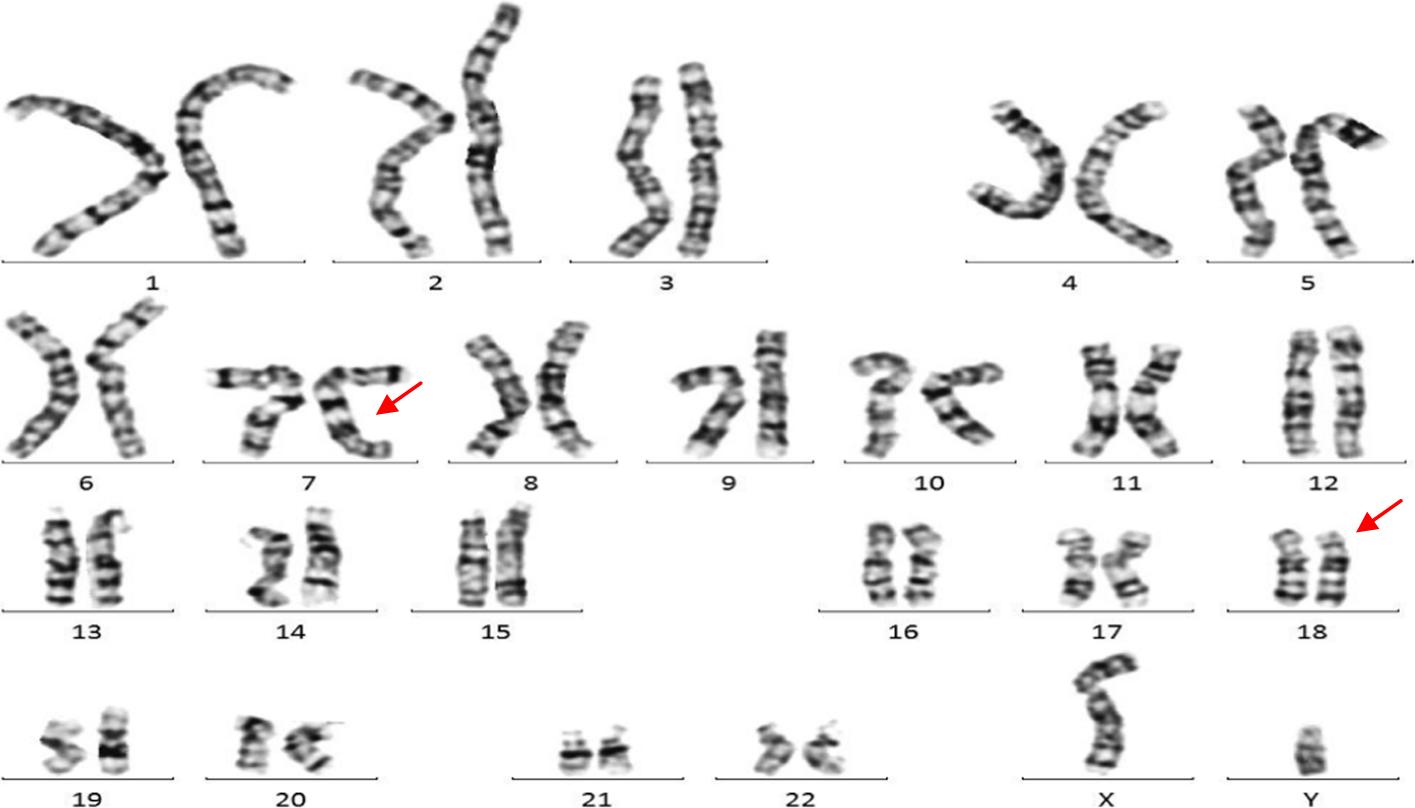
**GTG banded karyotype of the proband’s father.** The karyotype of the father indicated an abnormal karyotype [46,XY, t(7;18) (q3?6; p11.2)].

The array-CGH analysis results of the proband highly indicated that the unbalanced translocation could be inherited from a balanced translocation carrier parent. To trace the origin of the translocation, cytogenetic analyses were provided to the proband’s parents, even though they had received cytogenetic analyses before at another hospital and revealed apparent normal karyotypes. With the guidance of array-based CGH results of the proband, the cytogenetic analyses revealed an abnormal karyotype [46,XY, t(7;18) (q36; p11.2)] of the proband’s father (Figure 5), and a normal karyotype [46,XX] of the proband’s mother. The cytogenetics suggested that the father of the proband was a carrier of the balanced translocation.

## Discussion and conclusions

It was reported that parent with balanced translocation has a 50% chance to deliver chromosomes with unbalanced translocation to proband, and it could be difficult to notice until the proband was born. Unlike previous report [11], we showed another case that the proband carried an unbalanced translocation inherited from a balanced translocation carrier parent, which resulted in partial monosomy for 18p and partial trisomy for 7q. The 18p deletion spanned approximately 14 Mb and the 7q duplication about 11.2 Mb, which meant the derivative chromosome was 2.8 Mb shorter than chromosome 18. Because the derivative chromosome and the original chromosome 18 were similar in size, and the replacement region 7q36.1-q36.3 was also light stained like 18p11.32-p11.21 region in cytogenetic analysis of prenatal samples at resolutions of 350–400 bands, karyotyping did not reliably detect the anomalies, and led to the prenatal missed diagnosis of the case. The newborn infant was demonstrated with craniofacial dysmorphism and multiple malformations, and cytogenetic analysis of his peripheral blood sample was applied, indicating a possible abnormal karyotype, but the breakpoint and the deletion region were difficult to determine at resolutions of 450–500 bands. Array-based CGH analysis was applied to the proband, identified the chromosomal abnormalities and mapped those changes onto the genome sequence. On the basis of search of the genes in the region between 18p11.32 and 18p11.21 in the NCBI MapViewer, about 20 genes were identified including four important genes (Data not shown). Holoprosencephaly (HPE) severely affects the craniofacial development of the infants. TGFB-induced factor homeobox 1 (TGIF1) mutation, leading to the loss of function of TGIF1, promotes the pathogenesis of HPE in the mouse model [12]. Twisted gastrulation homolog 1 (TWSG1) has been demonstrated to be also associated with HPE [13,14]. Accordingly, TGIF1 and TWSG1 deficiency maybe important factor for the feature of craniofacial dysmorphism and skull joint separation of the proband. As for other two genes, THO complex is reported to influence the cerebellar congenital hypoplasia, Cell death-inducing DFFA-like effector a (CIDEA) is associated with fat and diabetes [15,16]. Thus, refined and complete diagnosis are needed to provide additional important manifestations information. Based on the specific genetic diagnosis of the affected infant, the clinical manifestations could also include mental retardation and growth retardation [17-19]. Accordingly, comprehensive genetic counseling and medical care were provided to the family for better preparation for the health and educational needs of the neonate.

In review of the case, several overlooked points leading to the missed diagnosis should be discussed and certain quality control strategies should be adopted to avoid similar problems in the future. Firstly, there was a past history of spontaneous abortions in the family, but POC samples were not analyzed. As indicated in the previous studies, spontaneous abortions affect 10–15% of all clinically recognized human pregnancies, and approximately 50% of first-trimester miscarriages are resulted from fetal chromosome abnormalities [20,21]. Cytogenetic analysis and (or) array-based CGH analysis of POC samples may provide valuable insights into the possible genetic causes of miscarriage and help predict the recurrence risk for subsequent pregnancies. Secondly, the couple has received cytogenetic analyses after spontaneous abortions at another hospital, but karyotyping failed to detect the balanced translocation. As estimated, balanced chromosomal rearrangements represented in about 0.19% in the general population, and it is common to observe decreased fertility or high rates of miscarriage in them [22]. Karyotyping is just able to detect a portion of the chromosomal rearrangements, because of its limited-resolution. Array-based CGH analysis of POC samples contributes to reveal submicroscopic chromosomal abnormalities, and provides valuable insights into the possible genetic causes of miscarriage [21,23]. Thirdly, when prenatal ultrasound examination of the fetus showed abnormal findings, only routine analysis of fetal chromosomes was applied, which failed to identify the unbalanced translocation and led to the prenatal missed diagnosis of the case. As prior studies suggested, about 71% of the clinically significant copy number alterations (CNAs) are smaller than 10 Mb in size, and are unlikely to be detected by routine analysis of fetal chromosomes [24]. Array-based CGH analysis possesses a number of significant advantages over conventional cytogenetic and other molecular cytogenetic techniques, and can enhance the detection rate by 10 to 16 percent of pregnancies with fetal ultrasound anomalies but not detected by routine analysis of fetal chromosomes [1,25-27]. The American Congress of Obstetricians and Gynecologists (ACOG) and the Society of Maternal Fetal-Medicine (SMFM) have recommended prenatal chromosomal microarray analysis as a first-line test in the case of abnormal ultrasound findings. Array-based CGH and karyotyping techniques are complemented by diverse detection spectrums and resolutions, and a combination of these methods can contribute to provide optimal genetic diagnosis and facilitate comprehensive medical care, as well as accurate recurrence risk counseling for the family. Given that the array-CGH analysis will not introduce additional risk for patients, it is reasonable to recommend that women with fetuses showing sonographic anomalies or de novo chromosome abnormalities already undergoing invasive testing should take the array-CGH test as an adjunct to conventional cytogenetic tests and molecular cytogenetic analysis [7,28].

## Consent

Written informed consent was obtained from the parents of the proband for publication of this Case report and any accompanying images.

## Competing interests

The authors declare that they have no competing interests.

## Authors’ contributions

XZ and AY defined the research theme. AY, JL, CL, LG, JW, MM and YZ described the case, performed the experimental work and organized the data. XZ and AY designed experiments, interpreted data, and drafted the manuscript. XZ critically reviewed the manuscript and provided concepts. All authors read and approved the final manuscript.
